# Selective Expression of KCNS3 Potassium Channel α-Subunit in Parvalbumin-Containing GABA Neurons in the Human Prefrontal Cortex

**DOI:** 10.1371/journal.pone.0043904

**Published:** 2012-08-24

**Authors:** Danko Georgiev, Guillermo González-Burgos, Mitsuru Kikuchi, Yoshio Minabe, David A. Lewis, Takanori Hashimoto

**Affiliations:** 1 Department of Psychiatry and Neurobiology, Kanazawa University Graduate School of Medical Science, Kanazawa, Ishikawa, Japan; 2 Department of Psychiatry, University of Pittsburgh, Pittsburgh, Pennsylvania, United States of America; 3 Research Center for Child Mental Development, Kanazawa University, Kanazawa, Ishikawa, Japan; 4 Department of Neuroscience, University of Pittsburgh, Pittsburgh, Pennsylvania, United States of America; Rikagaku Kenkyūsho Brain Science Institute, Japan

## Abstract

The cognitive deficits of schizophrenia appear to be associated with altered cortical GABA neurotransmission in the subsets of inhibitory neurons that express either parvalbumin (PV) or somatostatin (SST). Identification of molecular mechanisms that operate selectively in these neurons is essential for developing targeted therapeutic strategies that do not influence other cell types. Consequently, we sought to identify, in the human cortex, gene products that are expressed selectively by PV and/or SST neurons, and that might contribute to their distinctive functional properties. Based on previously reported expression patterns in the cortex of mice and humans, we selected four genes: KCNS3, LHX6, KCNAB1, and PPP1R2, encoding K^+^ channel Kv9.3 modulatory α-subunit, LIM homeobox protein 6, K^+^ channel Kvβ1 subunit, and protein phosphatase 1 regulatory subunit 2, respectively, and examined their colocalization with PV or SST mRNAs in the human prefrontal cortex using dual-label in situ hybridization with ^35^S- and digoxigenin-labeled antisense riboprobes. KCNS3 mRNA was detected in almost all PV neurons, but not in SST neurons, and PV mRNA was detected in >90% of KCNS3 mRNA-expressing neurons. LHX6 mRNA was detected in almost all PV and >90% of SST neurons, while among all LHX6 mRNA-expressing neurons 50% expressed PV mRNA and >44% expressed SST mRNA. KCNAB1 and PPP1R2 mRNAs were detected in much larger populations of cortical neurons than PV or SST neurons. These findings indicate that KCNS3 is a selective marker of PV neurons, whereas LHX6 is expressed by both PV and SST neurons. KCNS3 and LHX6 might be useful for characterizing cell-type specific molecular alterations of cortical GABA neurotransmission and for the development of novel treatments targeting PV and/or SST neurons in schizophrenia.

## Introduction

The core features of schizophrenia include disturbances in diverse cognitive functions that depend on the neural circuitry of the cerebral cortex [1]. In the cortex of subjects with schizophrenia, inhibitory neurotransmission mediated by γ-aminobutyric acid (GABA) appears to be altered [2], as indicated by lower levels of the mRNAs encoding the 67 kilodalton isoform of glutamic acid decarboxylase (GAD67) [3], the enzyme principally responsible for GABA synthesis, and the GABA membrane transporter 1 (GAT1) [4], [5], [6], [7], [8], which mediates the reuptake of synaptically released GABA. These alterations appear to involve specific subsets of GABA neurons. For example, the mRNAs encoding parvalbumin (PV) and somatostatin (SST), each of which is expressed in a separate subset of cortical GABA neurons, are decreased in schizophrenia [7], [9], [10], [11]. Moreover, the reductions in GAD67 and GAT1 appear to be prominent in PV- as well as SST-expressing GABA neuron subsets [6], [9], [10]. On the other hand, measures of the mRNA and protein for calretinin (CR), which is expressed by a third subset of GABA neurons, were unaltered in the cortex of subjects with schizophrenia [7], [9], [11], [12], [13], [14]. Together, these findings indicate that cortical dysfunction in schizophrenia selectively involves two separate subsets of GABA neurons: PV and SST neurons.

Understanding the molecular processes underlying the alterations in PV and SST neurons would be informed by identifying molecules that are selectively expressed in these neurons and that contribute to their distinctive functions. The evaluation of such molecules in schizophrenia might also reveal affected molecular pathways in PV and/or SST neurons, which could be used for developing therapeutic strategies targeting selectively these neurons. In order to identify such molecules, we first used published gene expression data for mouse cortical neuron subsets and selected 70 genes found to be either developmentally upregulated or preferentially enriched in PV and/or SST neurons [15], [16], [17]. We then evaluated the expression patterns of these 70 genes in the online atlases of gene expression in the mouse or human cortex [18] and excluded genes that were detected in pyramidal-like neurons with an apical dendrite, or that exhibited an apparently different laminar expression pattern from those of PV and/or SST mRNAs. We found that KCNS3, LHX6, KCNAB1 and PPP1R2 had cortical mRNA expression patterns similar to those of PV and/or SST mRNAs. KCNS3 encodes voltage-gated K^+^ channel Kv9.3 modulatory α-subunit that coassembles with Kv2.1 α-subunits and leads to an enhanced conductance and modified gating properties of the heteromeric channels [19], [20], [21]. LHX6 encodes LIM homeobox protein 6, a transcription factor suggested to be involved in the development of PV and SST neurons in the mouse cortex [22], [23]. KCNAB1 encodes K^+^ channel Kvβ1 accessory subunit that confers fast N-type inactivation to Kv1.1 channels [24]. PPP1R2 gene encodes protein phosphatase 1 (PP1) regulatory subunit 2, which inhibits PP1 and controls signal transduction and synaptic plasticity [25]. In this study, we determined whether KCNS3, LHX6, KCNAB1 and PPP1R2 mRNAs are selectively expressed in PV and/or SST neurons in the human prefrontal cortex (PFC).

## Materials and Methods

### Ethics Statement

All procedures were approved by the University of Pittsburgh Committee for Oversight of Research and Clinical Training Involving Decedents and Institutional Review Board for Biomedical Research as well as by the Ethics Committee of Kanazawa University Graduate School of Medical Science. All brain specimens were obtained during autopsy at the Allegheny County Medical Examiner’s Office (Pittsburgh, PA) after obtaining a witnessed verbal consent from the next of kin, using a procedure reviewed and approved by the University of Pittsburgh Committee for Oversight of Research and Clinical Training Involving Decedents. Verbal consent was audiotaped and a written document summarizing the consent process was generated and signed by the individual obtaining consent and an independent witness.

### Subjects

In this study we used post-mortem brain samples from four control subjects (two males and two females; 41 to 62 years of age) without a history of neurological or psychiatric diseases as determined by clinical records, interviews with relatives, toxicology results and neuropathological exams. Postmortem intervals and brain pH were within the ranges of 9.2–11.6 hours and 6.2–6.6, respectively, and RNA integrity numbers, which indicate the integrity of RNA in the tissue, were between 7.8 and 9.0, assuring their high quality for mRNA expression analyses [26].

### Tissue Preparation and Single-label in Situ Hybridization (ISH) with ^35^S-labeled Riboprobes

For each subject, the right PFC was blocked coronally, immediately frozen, and stored at –80°C. Serial sections (20 µm) containing the superior frontal gyrus were cut, thaw mounted onto glass slides, and stored at –80°C until processed. The location of PFC area 9 was identified by cytoarchitectonic criteria in Nissl-stained sections as described previously [27]. Templates for the synthesis of riboprobes were obtained by PCR using specific primer sets for the studied genes (Table S1). For LHX6 and KCNAB1, which have several splice isoforms, amplified fragments were located in the common region among all isoforms. Nucleotide sequencing revealed 100% homologies for all amplified fragments to the reported sequences in Genbank. These fragments were subcloned into the plasmid pSTBlue-1 (Novagen, Madison, WI). Antisense and sense probes were transcribed *in vitro* in the presence of ^35^S-CTP (PerkinElmer, Waltham, MA), using T7 or SP6 RNA polymerase (Promega, Madison, WI). The templates were then digested with RQ1 DNase (Promega), and riboprobes were purified by centrifugation through the RNeasy mini spin columns (Qiagen, Hilden, Germany). Hybridization was performed as described previously [8], [9], [10]. For each gene, we processed at least 2 sections per subject. After fixation with 4% paraformaldehyde in PBS, the sections were acetylated with 0.25% acetic anhydrate in 0.1 M triethanolamine/0.9% NaCl for 10 min, and dehydrated through a graded ethanol series. The sections were then hybridized with ^35^S-labeled riboprobes (2×10^7^ dpm/ml) in hybridization buffer containing 50% formamide, 0.75 M NaCl, 20 mM 1,4-piperazine diethane sulfonic acid, pH 6.8, 10 mM EDTA, 10% dextran sulfate, 5× Denhardt’s solution (0.2 mg/ml Ficoll, 0.2 mg/ml polyvinylpyrrolidone, 0.2 mg/ml BSA), 50 mM dithiothreitol, 0.2% SDS, and 100 µg/ml yeast tRNA at 56°C for 16 hr. The sections were washed in a solution containing 0.3 M NaCl, 20 mM Tris-HCl, pH 8.0, 1 mM EDTA, pH 8.0, and 50% formamide at 63°C, treated with 20 µg/ml RNase A (Sigma-Aldrich, St Louis, MO) at 37°C, and washed in 0.1×SSC (150 mM NaCl, 15 mM sodium citrate) at 66°C. Sections were then dehydrated through a graded ethanol series, air dried, and exposed to BioMax MR film (Kodak, Rochester, NY) for 5–10 days. After the exposure to film, sections were coated with NTB emulsion (Kodak) diluted 1∶1 with distilled water. To ensure the consistency of emulsion thickness across sections, slides were dipped by using a mechanical dipper (Aiden, Kobe, Japan), at a constant withdrawal speed (5 mm/s for KCNAB1, 10 mm/s for LHX6 and PPP1R2, 15 mm/s for KCNS3) and temperature (43°C). Sections were exposed for 7 days at 4°C, developed with D-19 (Kodak), and counterstained with 0.5% cresyl violet (Sigma-Aldrich).

### Film Autoradiographic Detection of mRNA Expression

Trans-illuminated autoradiographic film images were captured by a video camera, digitized, and analyzed using a Microcomputer Imaging Device (MCID) system (InterFocus Imaging Ltd, Cambridge, UK). Images of adjacent sections stained with cresyl violet were also captured and superimposed onto the autoradiographic images to draw contours of pia mater and gray matter/white matter border, and to denote the cortical layers. Optical density was expressed as microcuries per gram of tissue (µCi/g) by reference to Carbon-14 radioactive standards (ARC Inc., St. Louis, MO) exposed on the same film.

**Figure 1:**
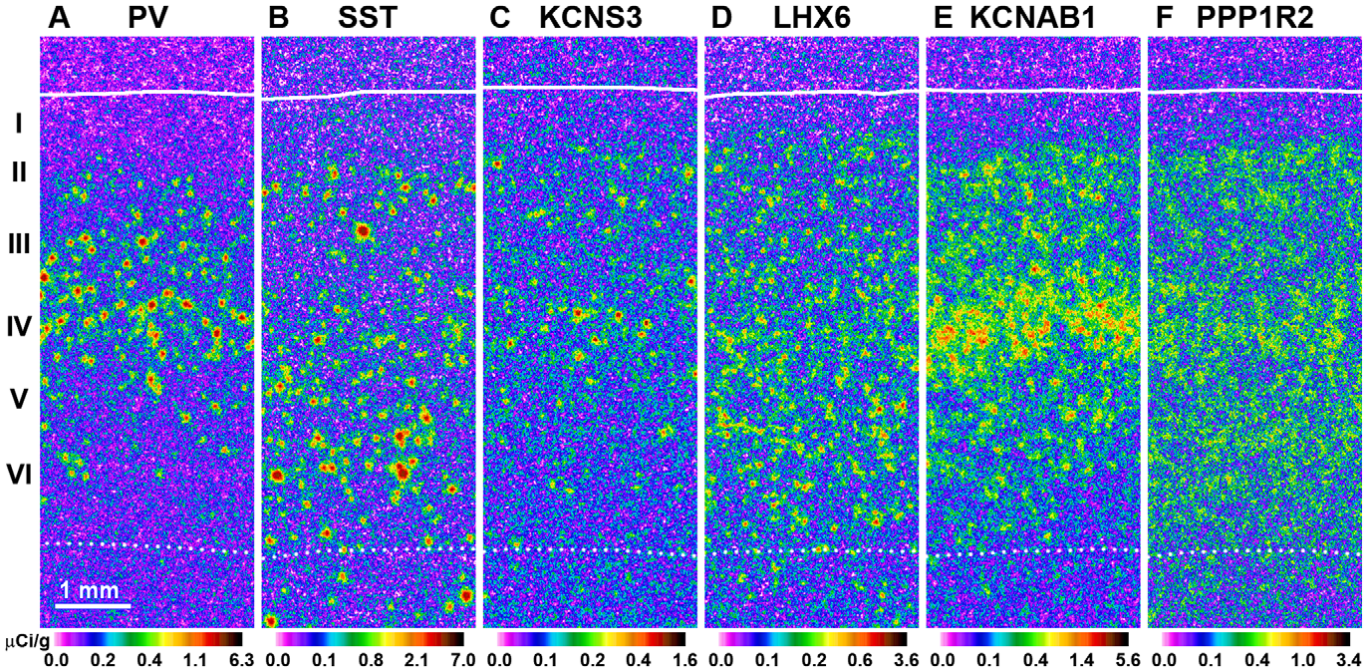
PV, SST, KCNS3, LHX6, KCNAB1 and PPP1R2 mRNA expression in the human PFC. Each mRNA was detected with a specific ^35^S-labeled antisense riboprobe. Signal intensity (µCi/g) for each mRNA in the film autoradiograms is represented in a pseudocolor manner according to the corresponding color scales. Roman numerals indicate the cortical layers, continuous white line indicates pia mater and a dashed line indicates the white matter/gray matter border.

### Identification of Specifically Labeled Neurons by ^35^S-labeled Riboprobes for KCNS3, LHX6, KCNAB1 and PPP1R2 mRNAs

For the identification of specifically labeled neurons in the following dual-label ISH analysis, a preparatory grain analysis was performed using the sections that were processed for single-label ISH and counterstained with cresyl violet. For each mRNA, two 0.4-mm-wide stripes spanning the whole thickness of the gray matter were sampled in at least one section from each subject. Because RNase A treatment of sections destroyed Nissl-stainable substances within the cytoplasm and made it difficult to draw contours of neuronal soma, we counted grains within circles with a fixed size of 22 µm diameter that covered the largest cross-sectional area of GABA neurons (≈400 µm^2^) observed in previous studies [27]. Using the MCID system equipped with a microscope, we captured bright- as well as dark-field images of the same views covering the entire cortical stripes at high magnification. In the bright-field images, the circles were centered over every Nissl-stained neuronal nucleus and over glial nuclei that were not immediately adjacent to an accumulation of grains (grain cluster). Small glial nuclei, stained darkly with cresyl violet, were easily discriminated from large and faintly stained neuronal nuclei (Figure S1). In the corresponding dark-field images, the numbers of grains were counted within each circle. Total numbers (mean ± SD across four subjects) of counted neurons were 1958 (490±60), 1675 (419±38), 2524 (631±73), and 2675 (669±57) and of counted glial cells were 1845 (461±55), 1009 (252±29), 1494 (374±42), and 1145 (286±23) for KCNS3, LHX6, KCNAB1 and PPP1R2 mRNAs, respectively. For each mRNA, a histogram of grain number per neuron (log_10_ transformed) for all sampled neurons revealed a distribution that appeared bimodal, presumably representing the modes of unlabeled and specifically labeled neuron populations [28] (Figure S1). Similar histograms of grain number per glial cell showed unimodal distribution for all mRNAs, representing the unlabeled cellular population with background grain numbers (Figure S1). In our dual-label ISH analysis without the counterstaining, glial nuclei cannot be visualized for the determination of background grain densities. Therefore, in each section, we determined the number of grains in a total of >0.1 mm^2^ cortical regions that do not contain any grain clusters and calculated the gray matter background grain number per 22-µm-diameter circle. For all studied mRNAs, a threshold of 5× background provided a cutoff that excluded glial cells and permitted the identification of specifically-labeled neurons (Figure S1). Furthermore, the 5× background thresholds were close to the point of rarity in the observed bimodal distributions that represented both labeled and non-labeled neurons (Figure S1). In a number of previous ISH studies using emulsion autoradiography, the criterion of ≥5× background was successfully used to define specifically labeled cells [9], [27], [29], [30].

**Figure 2:**
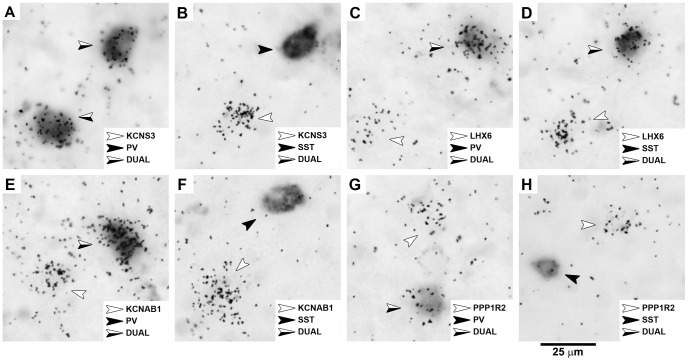
KCNS3, LHX6, KCNAB1 and PPP1R2 mRNAs detected simultaneously with PV or SST mRNAs. The signals for each of KCNS3, LHX6, KCNAB1 and PPP1R2 mRNAs were detected by ^35^S-labeled antisense riboprobes as silver grain clusters (white arrows), while signals for PV or SST mRNAs were detected by DIG-labeled antisense riboprobes as color reaction products (black arrows). Cells detected by both ^35^S- and DIG-labeled antisense riboprobes are shown with half white half black arrows.

**Figure 3:**
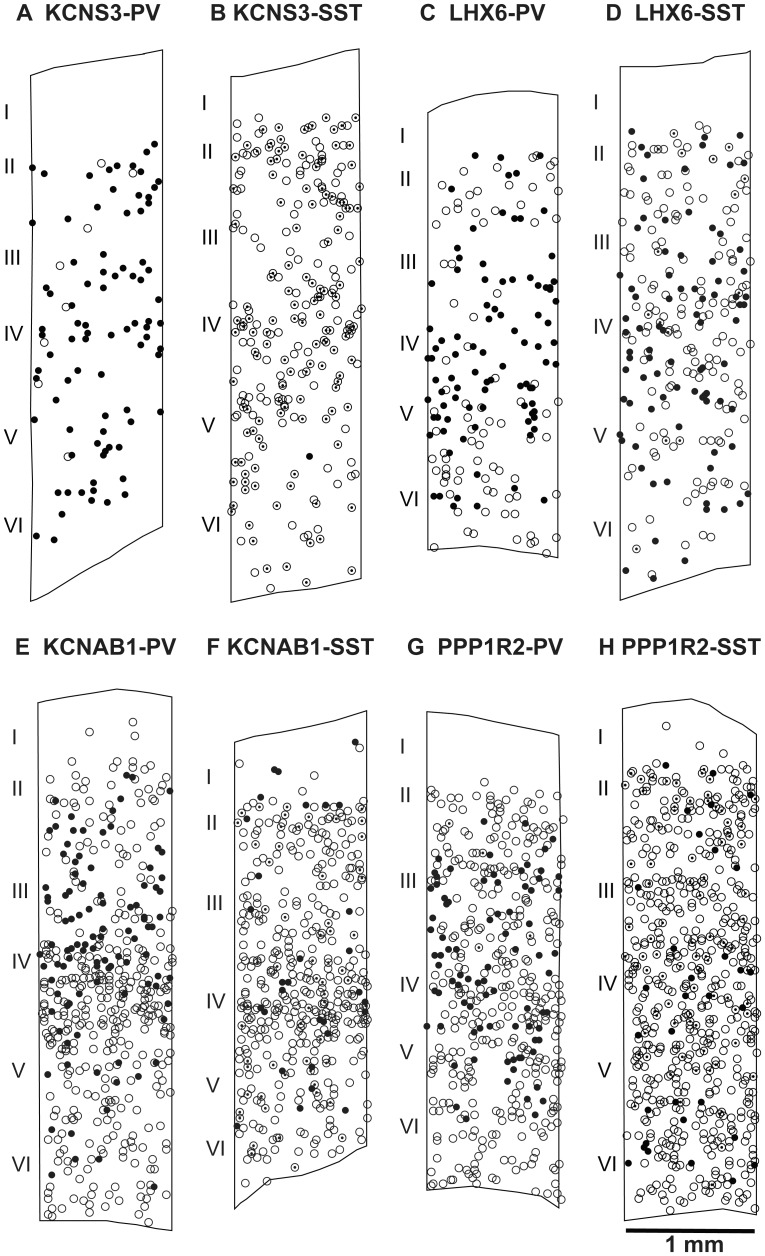
Cortical distributions of single- and dual-labeled cells. Neurolucida plots of single- and dual-labeled cells in representative cortical stripes for each of the eight dual-label ISH experiments performed with the combination of one ^35^S-labeled antisense riboprobe for KCNS3, LHX6, KCNAB1 or PPP1R2 mRNAs and one DIG-labeled antisense riboprobe for PV or SST mRNAs. ^35^S-riboprobe single-labeled cells are shown with white circles, DIG-riboprobe single-labeled cells are shown with circled dots, and dual-labeled cells are shown with black circles.

**Table 1:** Total numbers of counted single- and dual-labeled neurons and relative percentages† of dual-labeled neurons.

Neurons detected with ^35^S-labeled riboprobe	Neurons detected with DIG-labeled riboprobe	Neurons detected with both riboprobes	Percentage of dual-labeled neurons among all neurons detected with ^35^S-labeled riboprobe	Percentage of dual-labeled neurons among all neurons detected with DIG-labeled riboprobe
KCNS3 (1452)	PV (1323)	KCNS3&PV (1322)	KCNS3&PV/KCNS3 (91.0±1.3%)	KCNS3&PV/PV (99.9±0.2%)
KCNS3 (1187)	SST (1239)	KCNS3&SST (19)	KCNS3&SST/KCNS3 (1.6±0.3%)	KCNS3&SST/SST (1.5±0.3%)
LHX6 (2433)	PV (1218)	LHX6&PV (1217)	LHX6&PV/LHX6 (50.0±1.5%)	LHX6&PV/PV (99.9±0.2%)
LHX6 (2406)	SST (1194)	LHX6&SST (1080)	LHX6&SST/LHX6 (44.9±2.7%)	LHX6&SST/SST (91.0±6.7%)
KCNAB1 (4687)	PV (1161)	KCNAB1&PV (1161)	KCNAB1&PV/KCNAB1 (25.3±3.3%)	KCNAB1&PV/PV (100%)
KCNAB1 (4046)	SST (1054)	KCNAB1&SST (253)	KCNAB1&SST/KCNAB1 (6.1±1.0%)	KCNAB1&SST/SST (23.5±4.3%)
PPP1R2 (6393)	PV (1304)	PPP1R2&PV (1290)	PPP1R2&PV/PPP1R2 (20.4±4.3%)	PPP1R2&PV/PV (98.9±0.7%)
PPP1R2 (6110)	SST (1210)	PPP1R2&SST (426)	PPP1R2&SST/PPP1R2 (6.9±1.0%)	PPP1R2&SST/SST (34.9±7.9%)

† Percentages are given as mean ± SD across the four control subjects.

### Dual-label ISH

To directly assess colocalization of each of KCNS3, LHX6, KCNAB1 and PPP1R2 mRNAs with PV or SST mRNAs, we performed dual-label ISH. Templates for the synthesis of PV and SST riboprobes were obtained by PCR using specific primer sets (Table S1). Antisense riboprobes transcribed from these fragments were shown to detect the specific signals of PV and SST mRNAs [8], [9], [10]. In addition to the ^35^S-labeled riboprobes for KCNS3, LHX6, KCNAB1 and PPP1R2 mRNAs, digoxigenin (DIG)-labeled riboprobes for PV and SST mRNAs were transcribed *in vitro* in the presence of DIG-11-UTP (Roche, Mannheim, Germany). Sections were treated as described above except for the combination of one of the four ^35^S-labeled probes and one of the two DIG-labeled probes at concentrations of 2×10^7^ dpm/ml and 100 ng/ml, respectively, in hybridization buffer. After the posthybridization washing, sections were preincubated with 3% BSA in 100 mM Tris-HCl, pH 7.5, 150 mM NaCl, 0.05% Triton X-100 for 30 min, incubated with anti-DIG antibody conjugated with alkaline phosphatase (Roche) diluted 1∶2000 in 1% BSA, 100 mM Tris-HCl, pH 7.5, 150 mM NaCl, 0.05% Triton X-100 for 12 hr at 4°C, washed in 100 mM Tris-HCl, pH 7.5, 150 mM NaCl and air dried. To detect KCNS3, LHX6, KCNAB1 or PPP1R2 mRNAs, sections were coated with NTB emulsion and developed as described above. For the detection of PV or SST mRNAs, sections were incubated in 0.5 mg/ml nitroblue tetrazolium and 0.19 mg/ml bromo-chloroindolylphosphate (Roche) in 100 mM Tris-HCl, pH 9.5, 100 mM NaCl, 50 mM MgCl_2_ for 24 hr. In dark-field images, circles of 22 µm diameter were centered onto visually identified grain clusters and those which contained grains ≥5× background (measured for each section in the gray matter) were considered specifically labeled for KCNS3, LHX6, KCNAB1 or PPP1R2 mRNAs. In bright-field images, DIG-riboprobe positive cells were distinguished by the precipitation of alkaline phosphatase-produced color product in the cell body. We have analyzed one section per each of the four subjects for each of the eight possible combinations of ^35^S-labeled and DIG-labeled riboprobes. For each section, single- and double-labeled cells were plotted by Neurolucida 9.0 (MBF Bioscience, Williston, VT) and counted separately in three 1-mm-wide cortical stripes. The percentages of dual-labeled cells within a given neuron subset were reported as mean ± SD across the four subjects.

## Results

### Cortical Expression of KCNS3, LHX6, KCNAB1 and PPP1R2 mRNAs

Single-label ISH with ^35^S-labeled antisense riboprobes revealed specific laminar patterns of PV, SST, KCNS3, LHX6, KCNAB1 and PPP1R2 mRNA expression in the human PFC (Figure 1). In emulsion-coated slides observed at high magnifications, grain clusters for all mRNAs were observed over neuronal nuclei, but not glial nuclei, indicating that the majority of positive cells are neurons (Figure S1). Several additional lines of evidence confirmed the specificity of the riboprobes for KCNS3, LHX6, KCNAB1 or PPP1R2 mRNAs. First, the laminar distributions of KCNS3, LHX6 and KCNAB1 mRNAs were consistent with previously reported distributions in the rodent and human cortex [18], [22], [31], [32]. Second, for all four mRNAs, accumulations of silver grains over neuronal and not glial nuclei were consistent with their reported expression in cortical neurons [16], [17]. Third, sense riboprobes for KCNS3, LHX6, KCNAB1 or PPP1R2 mRNAs did not detect any signals above background (data not shown). The specificities of the PV and SST riboprobes were demonstrated in our previous studies [8], [9], [10].

Similar to the expression pattern of PV mRNA (Figure 1A), KCNS3 mRNA signals were high in layers II to V, with the highest signal density in layer IV (Figure 1C). The signals for LHX6 mRNA were moderate in layers II-III and higher in layers IV–VI, and some LHX6 mRNA signals were detected in the white matter just below the gray matter (Figure 1D). This distribution pattern appeared to correspond to the combined distribution of PV and SST mRNAs (Figures 1A and B). KCNAB1 mRNA signal densities were high throughout layers II-VI with a prominent accumulation in layer IV and superficial layer V (Figure 1E). PPP1R2 mRNA signals were detected almost evenly across layers II–VI with some accumulation in layer IV (Figure 1F). No specific hybridization signal was detected in layer I for any of the mRNAs studied.

### KCNS3, LHX6, KCNAB1 and PPP1R2 mRNA Expression in PV and SST Neurons

In order to determine if KCNS3, LHX6, KCNAB1 and PPP1R2 mRNAs were colocalized with PV or SST mRNAs, we performed dual-label ISH with a ^35^S-labeled antisense riboprobe for one of KCNS3, LHX6, KCNAB1 and PPP1R2 mRNAs and a DIG-labeled antisense riboprobe for PV or SST mRNAs. KCNS3, LHX6, KCNAB1 and PPP1R2 mRNAs were detected by accumulations of silver grains generated in emulsion layer by the corresponding ^35^S-labeled riboprobe. The neurons that express each of these mRNAs were identified as grain clusters with grain densities ≥5× background within 22 µm-diameter circles centered on each cluster (see Materials and Methods), whereas the neurons expressing either PV or SST mRNAs were visualized by color reaction of alkaline phosphatase that was conjugated to anti-DIG antibodies (Figure 2). Neurons detected with either a ^35^S-labeled, a DIG-labeled, or both ^35^S-labeled and DIG-labeled riboprobes were independently plotted and counted within three 1-mm-wide cortical stripes per subject, each traversing from the pial surface through the entire thickness of cortex (Figure 3). Quantitative data of the numbers of labeled neurons for each combination of ^35^S- and DIG-labeled riboprobes are summarized in Table 1.

Grain clusters representing KCNS3 mRNA-expressing neurons were observed over 99.9±0.2% of PV neurons, and 91.0±1.3% of the KCNS3 mRNA-positive grain clusters were labeled for PV mRNA (Figures 2A and 3A, Table 1). In contrast, only 1.5±0.3% of SST neurons contained grain clusters for KCNS3 mRNA, and 1.6±0.3% of such grain clusters were colocalized with SST mRNA (Figures 2B and 3B, Table 1). These data indicate that KCNS3 mRNA is specifically expressed by virtually all PV neurons. In addition, the absence of KCNS3 mRNA in the majority of SST neurons was consistent with the non-overlapping expression of PV and SST in cortical GABA neurons [33], [34].

Grain clusters representing LHX6 mRNA-expressing neurons were observed over 99.9±0.2% of PV neurons. However, 50.0±1.5% of LHX6 mRNA-expressing neurons were labeled for PV mRNA (Figures 2C and 3C, Table 1). Similarly, grain clusters for LHX6 mRNA-expressing neurons were observed over the majority (91.0±6.7%) of SST neurons, whereas 44.9±2.7% of LHX6 mRNA expressing neurons were labeled for SST mRNA (Figures 2D and 3D, Table 1). Given that PV and SST neurons represent non-overlapping cortical GABA neuron subsets [33], [34], these data demonstrate that nearly all (>94%) of LHX6 mRNA-expressing neurons are accounted for by the combination of PV and SST neurons.

Grain clusters representing KCNAB1 mRNA-expressing neurons were observed over all PV neurons and 23.5±4.3% of SST neurons. However the majority (>68%) of grain clusters for KCNAB1 mRNA-expressing neurons did not have signals for PV or SST mRNAs (Figures 2E, F and 3E, F, Table 1). Grain clusters representing PPP1R2 mRNA-expressing neurons were observed over 98.9±0.7% PV neurons and 34.9±7.9% of SST neurons. However, the majority (>72%) of grain clusters for PPP1R2 mRNA-expressing neurons did not have signals for PV or SST mRNAs (Figures 2G, H and 3G, H, Table 1). These findings indicate that KCNAB1 and PPP1R2 mRNAs were expressed by much larger populations of cortical neurons than PV or SST neurons.

## Discussion

In dual-label ISH, KCNS3 mRNA was detected in almost all PV neurons, but not in SST neurons, and PV mRNA was detected in >90% of KCNS3 mRNA-expressing neurons. LHX6 mRNA was detected in almost all PV and >90% of SST neurons, while among all LHX6 mRNA-expressing neurons 50% expressed PV mRNA and >44% expressed SST mRNA. KCNAB1 and PPP1R2 mRNAs were detected in much larger populations of cortical neurons than PV or SST neurons. Thus, KCNS3 mRNA appears to be a selective marker of PV neurons, whereas LHX6 mRNA is expressed almost exclusively by both PV and SST neurons. Only minor fractions of KCNS3 mRNA-expressing neurons (<8%) and LHX6 mRNA-expressing neurons (<6%) do not appear to express either PV or SST mRNAs (Table 1). These neurons might overlap with other minor subsets of cortical neurons [33].

For assessing coexpression of two molecules in single cells, ISH detection of mRNAs has several advantages over the detection of proteins by immunohistochemistry (IHC). First, because of predominant somal expression of most mRNAs, ISH can demonstrate directly overlapping signals for two mRNAs in the soma. On the other hand, differences in subcellular distribution of certain proteins and polypeptides among neuronal subtypes may mask the detection of their expression by IHC in certain cell types. For example, the majority of cholecystokinin (CCK) immunoreactive somata in the cortex were shown to be GABA neurons, but not pyramidal neurons [35]. However, CCK mRNA could be easily detected in the pyramidal neuron somata with ISH [36]. This discrepancy was explained by the predominant accumulation of CCK protein in the axon terminals, but not in the somata, of pyramidal neurons [37]. Second, ISH with ^35^S-labeled riboprobes has a high sensitivity that enables the detection of as few as 10 mRNA copies per cell [38], whereas the sensitivity of IHC, which depends on the specificity of antibodies, might not be high enough to identify cells containing low levels of target proteins. Third, in ISH with emulsion autoradiography, specifically labeled cells are identified using quantitatively determined cut-offs based on the distribution of grain numbers per cell, whereas in IHC, identification of positive cells tends to depend on visual impression which is less reliable for cells with weak immunopositive signals. These methodological factors might explain why PPP1R2 was demonstrated to be selective to PV neurons by IHC in the mouse cortex [17], but in our ISH study, PPP1R2 mRNA was detected in a larger neuronal population than PV neurons. Although the types of cortical neurons that express PPP1R2 might differ between mouse and human, the absence of protein signals and the presence of mRNA for PPP1R2 in many non-PV neurons might reflect a subcellular localization or the low expression levels of PPP1R2 protein in non-PV neurons.

LHX6, which encodes a LIM homeobox transcription factor, regulates the development of PV and SST neurons, both of which originate from the medial ganglionic eminence (MGE) [22], [23], [31], [39], [40]. In LHX6 knockout mice, the tangential migration of GABA neuron progenitors from the MGE to the cortex was delayed and the subsequent radial migration of these progenitors within the cortical plate appeared to be disturbed, resulting in an abnormal cortical distribution of GABA neurons [22], [23]. The knockout mice also exhibited severely reduced numbers of PV and SST neurons, indicating that LHX6 is important for the phenotypic differentiation of these neurons [22], [23]. Furthermore, the persistent expression of LHX6 after development suggests its role in the maintenance of physiological and morphological properties of mature PV and SST neurons [16], [31]. We recently reported decreased LHX6 mRNA levels in the PFC of subjects with schizophrenia [14]. Interestingly, a subset of schizophrenia subjects was identified by the presence of consistent deficits in LHX6, GAD67, PV and SST mRNAs, suggesting that LHX6 deficits may affect development and/or maintenance of GABAergic phenotype in PV and SST neurons in these subjects. Identifying genes that are regulated by LHX6 may help inform molecular mechanisms of the alterations in PV and SST neurons in schizophrenia.

KCNS3 mRNA, which is expressed selectively by the majority of PV neurons in the PFC, encodes Kv9.3 voltage-gated K^+^ channel modulatory α-subunit. Modulatory α-subunits do not assemble into homomeric channels, but selectively associate with delayed rectifier Kv2 subunits to form functional heteromeric channels [41]. In heterologous expression systems, Kv9.3 subunits coassemble with Kv2.1 subunits, with a 1∶3 subunit stoichiometry, to form heteromeric Kv2.1/Kv9.3 channels [21]. Kv2.1 immunoreactivity has been detected in the soma and dendrites of most cortical neurons, including PV neurons [42], [43]. Therefore, KCNS3 mRNA expression in PV neurons indicates the presence of heteromeric Kv2.1/Kv9.3 channels in the soma and dendrites of these neurons. Compared with homomeric Kv2.1 channels, Kv2.1/Kv9.3 channels have modified characteristics, such as larger single channel conductance, faster activation, slower deactivation and inactivation, and shifted steady-state activation and inactivation curves towards more negative values by ≈20 mV [19], [20]. Thus, Kv2.1/Kv9.3 channels are more effectively activated by subthreshold membrane depolarizations such as those generated by excitatory synaptic inputs. Interestingly, a recent electrophysiological and modeling study demonstrated that voltage-dependent K^+^ currents activated by excitatory post-synaptic potentials (EPSPs) in PV neuron dendrites shorten both the time course of EPSPs and the time window for the summation of multiple EPSPs produced by excitatory synaptic inputs at spatially separated dendritic sites [44]. Therefore, the presence of Kv9.3 subunits might contribute to the fast excitatory synaptic transmission and precise detection of coincident synaptic inputs in PV neurons [45]. Kv3 channels, which are essential for the ability of PV neurons to fire a train of short-duration action potentials at high frequencies [46], were also implicated in fast EPSPs and narrow time window of EPSP summation in PV neurons [44]. However, Kv3 channels are activated only by membrane potentials more positive than approximately −10 mV that can be attained during action potentials, but not synaptic EPSPs [46]. Therefore, Kv3 channels are unlikely to play a central role in regulating time course and summation of EPSPs in PV neurons.

PV neurons play an essential role in the generation of cortical gamma oscillations [47], [48] that are important for cognitive processes [49]. Because fast excitatory synaptic transmission and precise coincident detection in PV neurons have been implicated in the generation of gamma oscillations [50], [51], our finding of selective KCNS3 expression in PV neurons suggests that KCNS3-encoded Kv9.3 subunits might contribute to the generation of gamma oscillations by PV neurons. In subjects with schizophrenia, alterations in GABA neurotransmission by PV neurons appear to contribute to cognitive deficits by altering gamma oscillations [3], [52]. Currently, no Kv2.1-selective pharmacological modulators are available [53] and, moreover, Kv2.1 subunits are ubiquitously expressed by cortical neurons [42], [43]. Therefore, the highly selective expression of KCNS3 mRNA in PV neurons suggests that Kv9.3 subunits might be a crucial target to modulate Kv2.1-mediated delayed rectifier K^+^ currents and thus neuronal activity in a PV neuron-selective manner. Pharmacological compounds targeting Kv9.3 subunits might be useful to directly assess the functional importance of Kv2.1/Kv9.3 currents in PV neurons and for developing therapeutic strategies selectively targeting PV neurons to modulate their role in the generation of gamma oscillations.

## Supporting Information

Figure S1 **Identification of neurons specifically labeled for KCNS3, LHX6, KCNAB1 and PPP1R2 mRNAs.** (A, C, E, G) High magnification view of Nissl-stained emulsion-coated sections hybridized with ^35^S-labeled antisense riboprobes for each of the four mRNAs (KCNS3, LHX6, KCNAB1 and PPP1R2). Silver grain clusters were observed over large and faintly stained neuronal nuclei, whereas small and more intensely stained glial nuclei have few scattered grains. N+: neuron labeled with ^35^S-labeled riboprobe, N-: neuron not labeled with ^35^S-labeled riboprobe; G: glial cell not labeled with ^35^S-labeled riboprobe. (B, D, F, H) Histograms showing the distribution of grain numbers per cell for neurons and glial cells. Arrows indicate the average 5× background grain number per cell across all Nissl-stained sections for each of the mRNAs.
(TIF)

Table S1 **Primer sets used for cloning of DNA templates.**
(DOC)
