# Supplementary figures and images for: Selective Expression of KCNS3 Potassium Channel α-Subunit in Parvalbumin-Containing GABA Neurons in the Human Prefrontal Cortex
Source: PLoS One. 2012 Aug 24;7(8):e43904. doi: 10.1371/journal.pone.0043904 (PMC3427167; doi:10.1371/journal.pone.0043904)

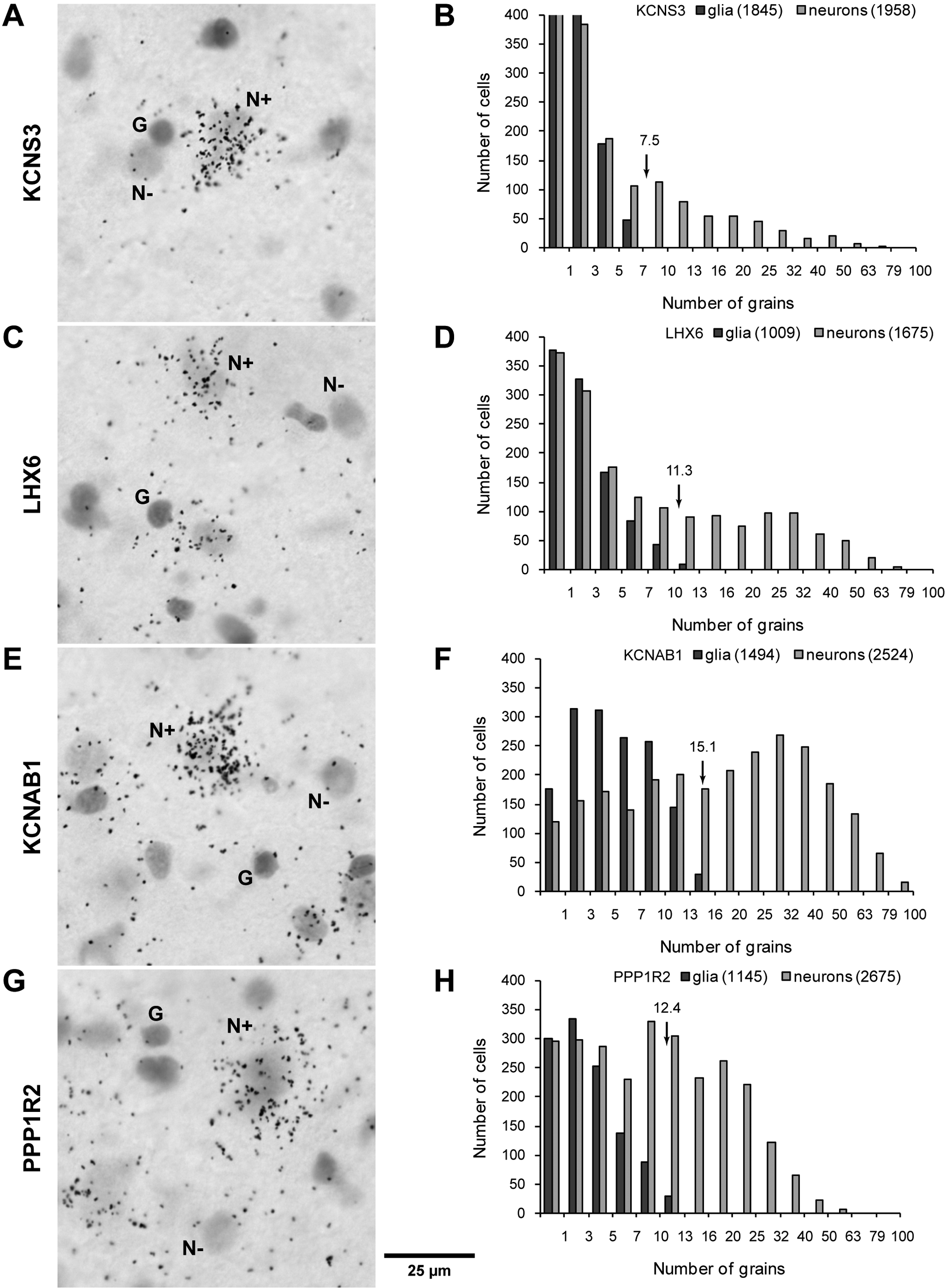

Supplement: Figure S1 — Identification of neurons specifically labeled for KCNS3, LHX6, KCNAB1 and PPP1R2 mRNAs. (A, C, E, G) High magnification view of Nissl-stained emulsion-coated sections hybridized with 35S-labeled antisense riboprobes for each of the four mRNAs (KCNS3, LHX6, KCNAB1 and PPP1R2). Silver grain clusters were observed over large and faintly stained neuronal nuclei, whereas small and more intensely stained glial nuclei have few scattered grains. N+: neuron labeled with 35S-labeled riboprobe, N-: neuron not labeled with 35S-labeled riboprobe; G: glial cell not labeled with 35S-labeled riboprobe. (B, D, F, H) Histograms showing the distribution of grain numbers per cell for neurons and glial cells. Arrows indicate the average 5× background grain number per cell across all Nissl-stained sections for each of the mRNAs. (TIF) [file pone.0043904.s001.tif]
