# Supplementary material for: Selective Expression of KCNS3 Potassium Channel α-Subunit in Parvalbumin-Containing GABA Neurons in the Human Prefrontal Cortex
Source: PLoS One. 2012 Aug 24;7(8):e43904. doi: 10.1371/journal.pone.0043904 (PMC3427167; doi:10.1371/journal.pone.0043904)
Supplement: Table S1 — Primer sets used for cloning of DNA templates. (DOC) [file pone.0043904.s002.doc]

**Table S1. Primer sets used for cloning of DNA templates.**

| **mRNA** | **GenBank** | **Forward primer** | **Reverse primer** | **Location** | **Product** |
| --- | --- | --- | --- | --- | --- |
| KCNS3 | NM_002252 | AGAATGGAGAATCCAGCGTACT | ATTCTCCAAGGAGGTGGTGTTA | 900-1841 † | 942 |
| LHX6 | NM_014368 | GACATATTGGACCAGAAGAGGC | CTGAGCTGTACTCACCACGTTC | 1880-2867 ‡ | 988 |
| KCNAB1 | NM_172160 | AGGTTGCTGAACGGCTGATGACC | TGTGCATCCCAGACGCTCCG | 420-1126 † | 707 |
| PPP1R2 | NM_006241 | GGAGCTCTAGGCCGGCGTCT | GGCTCAGGGTCGCTGCTTGG | 104-356 ‡ | 253 |
| PV | NM_002854 | GTCGATGACAGACTTGCTGAAC | GCAGTCAGTGCTTCTTAGCTTTC | 53-397 † | 345 |
| SST | NM_001048 | CCCAGACTCCGTCAGTTTCT | TCGCTGAAGACTTGGAGGAT | 207-562 † | 356 |

† The cloned fragment is located in the protein coding region of the mRNA.

‡ The cloned fragment is located in the non-coding region of the mRNA.
